# Cerebral Venous Thrombosis in the Mediterranean Area in Adults. Role of Behçet’s Disease as an Underlying Cause

**DOI:** 10.4084/MJHID.2011.044

**Published:** 2011-10-24

**Authors:** N.Y. Barlas, G. Akman-Demir, S.Z. Bahar

**Affiliations:** Istanbul Faculty of Medicine, Department of Neurology, Istanbul University

## Abstract

Cerebral venous and dural sinus thrombosis (CVT) is a rare condition with a wide spectrum of clinical presentations. The epidemiology of the disease has evolved considerably during the recent decades with increasing oral contraceptive use in young and middle-aged women. CVT has various causes including genetic and acquired prothrombotic disorders and it usually has a favorable outcome with a low rate of thrombotic recurrence and mortality. Geographical and ethnic variations between populations may result in different distribution of CVT etiologies leading to different pathophysiological mechanisms and clinical presentations. In CVT series reported mostly from the Americas and the western European countries Behçet’s disease (BD) is not reported as a common cause of CVT. However it can be discerned as a frequent cause of CVT in BD series. Due to the high prevalence of BD in the southeast Mediterranean region BD is a frequent cause of CVT in the area. Discerning characteristics of patients with BD and CVT have been reported previously and these might be helpful in guiding diagnosis and treatment of CVT especially in this part of the world.

Cerebral venous and dural sinus thrombosis (CVT) presents with a broad spectrum of symptoms, and it is caused by a variety of etiologies.^1^–^2^ Nearly 85% of CVT cases have a prothrombotic risk factor or a direct causative disorder. These include genetic and acquired prothrombotic disorders, cancer, hematological diseases, pregnancy and puerperium, systemic inflammatory diseases, neurosurgical procedures and some local anatomical causes such as ear, head or neck infections. Around 40% of CVT patients have more than one risk factor.^3^ Among the systemic inflammatory disorders, systemic lupus erythematotsus (SLE), antiphospholipid syndrome, sarcoidosis and Behcet’s disease (BD) can be listed.

CVT affects approximately 5 people per million annually.^4^ Although underlying factors may be common with other types of venous thromboembolism, the unique way the central nervous system is affected with CVT gives it an exceptional place under the concept of venous thrombosis. Even though CVT can develop in the course of various disorders, due to its clinical presentation most of the patients are evaluated within stroke cohorts. CVT accounts for 0.5 to 1% of all strokes.^2^,^4^ In Istanbul Medical School Stroke Registry CVT comprises 1% of all strokes.^5^ It is hard to estimate the number of CVT cases associated with various etiological processes that are not included in stroke registries. The largest information on CVT is provided by International Study on Cerebral Vein and Dural Sinus Thrombosis (ISCVT) comprised of 624 cases. In that prospective observational study, aimed to determine prognosis of CVT, 624 symptomatic CVT cases from 21 countries were registered over 3 years and ample information about all characteristics of CVT patients were obtained as well. Fifty-seven percent of these cases are from North-western Mediterranean countries (France, Portugal, Spain and Italy). Overall 74.5% of patients were women and in 2/3rds of them CVT was associated with pregnancy, puerperium, oral contraceptives (OC) or hormone replacement therapy (HRT); factors associated with gender. The second most common etiological factor was thrombophilia with a rate of 34%. However, these figures do not reflect the true underlying factor incidence since in more than 40 % of the cases there was more than one risk factor.

Information on epidemiology and risk factors for cerebral venous thrombosis (CVT) in North-western Mediterranean countries are largely available. On the contrary, information on CVT in the South-eastern Mediterranean region is relatively sparse. The aim of this review is to focus on CVT and inherited and acquired causes in countries of the South-eastern Mediterranean region.

Behcet’s disease is one of the systemic inflammatory diseases with high risk of venous thromboembolism and it is seen more commonly along the Silk Route that extends from the Mediterranean region to Japan.^6^ In the South-eastern Mediterranean region etiology of CVT differs compared to Western countries, due to the higher prevalence of Behcet’s disease in the region. Behcet’s disease is a systemic inflammatory disorder of unknown etiology, which presents with recurrent oral aphtae, genital ulcerations, and uveitis. Diagnostic criteria for BD are listed on Table 1.^7^ Central nervous system (CNS) involvement is seen about in 5–10% of cases with BD.^8^–^9^ Besides the more commonly encountered parenchymal neurological involvement which occurs mainly as a brainstem meningoencephalitis, intracranial hypertension due to CVT may be seen in about 15% of the patients with neurological involvement. In our series this ratio was 17%, in another series from Tunisia it was 11%.^10^–^11^

Although population based studies are lacking from South-eastern Mediterranean countries, a previous study comparing a series of CVT patients with and without BD revealed that more than half of the patients diagnosed with CVT have BD as the underlying factor.^12^ When the yearly rate of CVT patients are compared according to etiology in this cohort 42% of the patients had BD as the causative factor compared to all other etiologies complied as a separate group as the underlying conditions for the remaining 58%.^12^ One point deserves further clarification because in this study, non-BD group consisted largely of patients that were admitted to the hospital with CVT; however, there may be another group of patients who present with isolated ICH symptoms or only headache that were either not diagnosed as CVT or were diagnosed but managed in the emergency room due to relatively less serious clinical presentation and consequently were not included in the database.

Behcet’s disease comprises a significant portion of CVT cases from many Mediterranean countries, although the percentage varies according to the country, and the center reporting the series. A series from France analyzing isolated intracranial hypertension as the only sign of CVT found that these patients consisted as high as 37% of all CVT patients and 22% of them had BD as the underlying cause; however, this center is one of the dedicated centers to Behcet’s disease in the country. In this cohort other common etiological factors were coagulopathies, unknown etiology, oral contraceptive use and other inflammatory diseases.^13^ On the other hand, data from Europe depicts the rarity of BD as an etiological factor of CVT, there were only 6 patients with BD among 624 patients registered in the ISCVT.^3^

Interestingly, however, a small CVT series from Lebanon did not have any patients with BD, among 16 cases.^14^ This might be reflecting a selection bias, as discussed below.

In another CVT series including only patients with cortical or deep vein thrombosis, excluding patients with dural sinus thrombosis from Turkey, similar frequencies of etiologies to those reported from the West with few CVT patients with BD was reported.^15^ Albeit small in size, in this series the distribution of etiological factors causing CVT among hormonal factors, malignancy, genetic thrombophilia, infections, hematological disorders, vasculitis and interventions was more or less similar to the largest cohort reported from the rest of the world composed mostly of Nortwestern European countries.^3^,^15^ Most probably, the inclusion of patients with only cortical or deep vein thrombosis was the reason for this difference, supporting the clinical presentation differences in CVT patients with and without BD emphasized in the comparative study.^12^ In a previous study of venous thrombosis from Turkey, it was concluded that BD may be taking part together with other etiologies in most of the cases;^16^ in that case when another etiology is found BD might have been overlooked in some centers not so familiar with BD. On the other hand, our center is a nationwide tertiary center with a specialized multidisciplinary BD clinic and the frequency of CVT due to BD in our center may be over-represented due to a selection bias. However, the prevalence of BD was estimated as 42/10,000 (95% CI, 34–51/10,000) in Istanbul, Turkey, supporting the notion that Turkey has the highest prevalence rate of the disease in the world.^17^ Therefore it is not surprising to encounter BD as a frequent etiology in CVT in the series reported from our center in Istanbul. Furthermore, a recent review including CVT patients with BD mainly from countries in the Mediterranean region showed that CVT incidence in BD was about 3 per 1000 person years.^18^

As mentioned above ¾ of the patients in ISCVT were women and the most common risk factor was oral contraceptive use.^3^ However, in BD which is slightly more prevalent among males, serious organ involvement is significantly more frequent in males and this gender predisposition is also seen in cerebral venous thrombosis patients with BD.^12^ In the study where a direct comparison was made in CVT patients with and without BD, CVT due to BD was significantly more common among male patients. In the same study the mean age was 26 in the non-BD group and 39 in the BD group, resulting in a significant difference. However in the largest CVT series published mean age was 37 for CVT patients, comparable to that seen in BD patients with CVT.^3^

The rarity of acute presentation with neurological deficits and seizures in patients with BD may preclude the inclusion of these patients in other CVT series.^7^ In CVT clinical features differ according to the location and the extent of the occlusion in the sinuses and veins and the course of the underlying disease process. A slowly progressing dural sinus thrombosis that does not affect the cortical venous circulation may present with isolated headache and normal neurological examination findings. These patients may be misdiagnosed as pseudotumor cerebri. However an acute clinical presentation with focal neurological deficits and seizures can be readily diagnosed as CVT with appropriate imaging techniques. CVT in BD patients mostly presents with signs of isolated intracranial hypertension and venous infarction rarely develops.^12^ As a consequence in CVT due to BD patients usually have an insidious onset, they present usually with headache as the only symptom and the neurological examination is normal or only papilledema or lateral gaze paresis indicative of intracranial hypertension is found.

In the past, CVT used to be considered a condition with bad prognosis, but currently CVT is considered to be a relatively more benign condition with mortality rates below 10%.^19^–^23^ According to the limited follow-up data on CVT with and without BD there were no significant differences between the two groups on outcome.^12^ A greater tendency for recurrence was found in patients with BD.^12^ A cytopathological study on BD CVT cases is not available however former autopsy findings on pulmonary arterial thrombosis suggests an inflammatory process underlying the thrombosis.^24^ This finding may explain the chronic course of the thrombotic process in CVT due to BD leading to the mentioned clinical differences.

The treatment of CVT mainly comprises of anticoagulation, with subcutaneous fractionated heparin at the acute stage, and 6 months of oral anticoagulation, afterwards.^25^ However, since the thrombosis of BD is considered an inflammatory process rather than a procoagulant process,^26^ treatment of these cases may vary. Although treatment of CVT due to BD is still debated and Class I evidence is lacking^27^ our approach is to use only steroids, instead of anticoagulants, as also suggested by EULAR task force.^28^ In such cases we usually give 5 consecutive days of IV methylprednisolone followed by a slow oral taper.^29^ In recent years we also tend to add long-term azathioprine to prevent recurrences or other vascular complications that could be seen in those patients.^27^ In intractable cases or repetitive CVTs due to BD, anticoagulants may be added to steroids; but anticoagulants should never be used alone.^29^ It should be kept in mind that pulmonary aneurysms should be ruled out before initiating any anticoagulant treatment in patients with BD.

Due to the scarcity of data on CVT and inherited and acquired causes in countries of the south-eastern Mediterranean region a comparison with northwestern Mediterranean region is quite difficult to make. However, one report emphasized the occurrence of CVT due to BD in Turkey which deserves attention both in terms of the differences in demographic and clinical features and possibly the treatment options. Therefore, in patients with CVT from southeast Mediterranean region, BD should be kept in mind especially if the patient is male, and if no other risk factor can be identified. BD should always be questioned since multi-etiology cases are not very rare. A multi-national and multi-center prospective analysis of the CVT cases in south-east Mediterranean region seems to be worthwhile in the near future.

## Figures and Tables

**Table 1:** Diagnostic criteria for Behçet’s disease (International Study Group for Behçet’s Disease, 1990)

Reccurrent oral aphtae:	at least three times in a year
Plus any two of genital ulcerations:	active lesion or scar
skin lesions:	erythema nodosum, foliculitis, other ulcerations
eye involvement:	anterior or posterior uveitis, or retinal vasculitis
positive pathergy test:	skin hyper-reactivity to pinprick (sterile pustule formed in 24–48 h)
